# Experimental Reactivation of Pulmonary *Mycobacterium avium* Complex Infection in a Modified Cornell-Like Murine Model

**DOI:** 10.1371/journal.pone.0139251

**Published:** 2015-09-25

**Authors:** Seung Bin Cha, Bo Young Jeon, Woo Sik Kim, Jong-Seok Kim, Hong Min Kim, Kee Woong Kwon, Sang-Nae Cho, Sung Jae Shin, Won-Jung Koh

**Affiliations:** 1 Department of Microbiology, Institute for Immunology and Immunological Diseases, Brain Korea 21 PLUS Project for Medical Science, Yonsei University College of Medicine, Seoul, South Korea; 2 Department of Biomedical Laboratory Science, College of Health Sciences, Yonsei University, Wonju, South Korea; 3 Division of Pulmonary and Critical Care Medicine, Department of Medicine, Samsung Medical Center, Sungkyunkwan University School of Medicine, Seoul, South Korea; Public Health Research Institute at RBHS, UNITED STATES

## Abstract

The latency and reactivation of *Mycobacterium tuberculosis* infection has been well studied. However, there have been few studies of the latency and reactivation of *Mycobacterium avium* complex (MAC), the most common etiological non-tuberculous *Mycobacterium* species next to *M*. *tuberculosis* in humans worldwide. We hypothesized that latent MAC infections can be reactivated following immunosuppression after combination chemotherapy with clarithromycin and rifampicin under experimental conditions. To this end, we employed a modified Cornell-like murine model of tuberculosis and investigated six strains consisting of two type strains and four clinical isolates of *M*. *avium* and *M*. *intracellulare*. After aerosol infection of each MAC strain, five to six mice per group were euthanized at 2, 4, 10, 18, 28 and 35 weeks post-infection, and lungs were sampled to analyze bacterial burden and histopathology. One strain of each species maintained a culture-negative state for 10 weeks after completion of 6 weeks of chemotherapy, but was reactivated after 5 weeks of immunosuppression in the lungs with dexamethasone (three out of six mice in *M*. *avium* infection) or sulfasalazine (four out of six mice in both *M*. *avium* and *M*. *intracellulare* infection). The four remaining MAC strains exhibited decreased bacterial loads in response to chemotherapy; however, they remained at detectable levels and underwent regrowth after immunosuppression. In addition, the exacerbated lung pathology demonstrated a correlation with bacterial burden after reactivation. In conclusion, our results suggest the possibility of MAC reactivation in an experimental mouse model, and experimentally demonstrate that a compromised immune status can induce reactivation and/or regrowth of MAC infection.

## Introduction

Nontuberculous mycobacteria (NTM) are widely spread in environment which may cause pulmonary disease, skin and soft tissue infections, lymphadenitis, and disseminated disease among which, chronic pulmonary disease is the most commonly found in clinical condition [1, 2]. NTM lung diseases are becoming more prevalent worldwide [3, 4], and the most common etiological agent of MTM lung disease is *Mycobacterium avium* complex (MAC) which consists of *Mycobacterium avium* and *Mycobacterium intracellulare* [1, 2]. Since NTM are ubiquitous in the environment and frequently isolated from both soil and water, humans could occasionally encounter these organisms. Nevertheless, it is generally believed that NTM infections are effectively controlled by normal host defense mechanism [5]. Thus, it is believed that several unknown susceptibility factors might affect the healthy individuals to develop the NTM lung disease [6].

Although analogous to tuberculosis (TB) within several ways, the natural pathogenesis of NTM infection is unknown. Likewise, it is still on debate whether NTM lung disease develops soon after infection or, like TB, develops after a period of latency. In humans, NTM may cause both symptomatic disease and asymptomatic infection, and previous skin test studies suggested that a substantial proportion have had prior and presumably asymptomatic NTM infection [7, 8].

As well as TB, NTM disease has emerged as important infectious complications in patients receiving tumor necrosis factor (TNF) antagonists [9–11]. It was suggested that the development of TB after TNF antagonist therapy is a reactivation of latent TB infection (LTBI) caused by immunosuppression [12, 13]. Moreover, recent studies have reported that TNF antagonist therapy is also a predisposing factor for NTM infection [9–11]. Together, these findings raise the possibility of latent NTM infection. However, unlike TB, the concept of 'latent' NTM infection is controversial and there is no solid scientific evidence of latent NTM infection. Moreover, there have been no experimental studies on ‘latent’ NTM infection due to a lack of suitable animal models. The Cornell model was developed to investigate the LTBI using by a murine model, in which mice infected with *M*. *tuberculosis* are treated with anti-TB drugs, resulting in an absence of detectable bacilli by in organ culture [14, 15]. Reactivation of bacilli was spontaneously occurred from this culture-negative state following immunosuppression. Here, we investigated the possibility of reactivation of MAC infection using the Cornell-like murine model.

## Materials and Methods

### Ethics statement

All animal experiments were performed in accordance with the Korean Food and Drug Administration (KFDA) guidelines. The experimental protocols used in this study were reviewed and approved by the Ethics Committee and Institutional Animal Care and Use Committee (Permit Number: 2012-0072-2) of the Laboratory Animal Research Center at Yonsei University College of Medicine (Seoul, Korea). Carbon dioxide (CO_2_) was used for euthanasia.

### Mice

Specific pathogen-free 5- to 6-week-old female C57BL/6 mice were purchased from Japan SLC, Inc. (Shijuoka, Japan). Mice were maintained under barrier conditions in a BL-3 biohazard animal facility at the Yonsei University Medical Research Center with constant temperature (24±1°C) and humidity (50±5%). Animals were fed a sterile commercial mouse diet and provided with water *ad libitum* under standardized light-controlled conditions (each 12 hours of light and dark period). Mice were monitored daily, and none of mice exhibited any clinical symptom or illness during this experiment.

### Chemicals and reagents

Antibiotics (clarithromycin and rifampicin) and immunosuppressant agents (dexamethasone and sulfasalazine) were purchased from Sigma Chemical Co. (St Louis, MO, USA). Middlebrook 7H9 broth, 7H10 agar substrate and oleic acid-albumin-dextrose-catalase (OADC) were purchased from Difco Laboratories (Detroit, MI, USA) and Becton Dickinson (Sparks, MD, USA), respectively.

### Bacterial strains, cultures and preparation of mycobacterial single cell suspensions

A total of six MAC strains consisting of both type strains and clinical strains were used in this study. Briefly, two types of strains, *M*. *avium* (MAV) 104 and *M*. *intracellulare* (MI) ATCC 13950, were obtained from the American Type Culture Collection (ATCC, Manassas, VA). In addition, we used four clinical isolates, MAV SM #1, MAV SM #22, MI SM #30, and MI SM #42, which were recovered from patients who met the diagnostic criteria of NTM lung disease, according to the American Thoracic Society and Infectious Diseases Society of America guidelines [2] (Samsung Medical Center, Seoul, Korea) [16, 17]. All strains used in this study showed a smooth colony type with the exception of MAV SM#22, which displayed a rough colony morphotype. The identity of MAC isolates was identified by *rpoB* sequencing analysis, MAC multiplex PCR [18] and *hsp65* code sequevar analysis [19]. Susceptibility of each MAC strains was measured according to the Clinical and Laboratory Standards Institute (CLSI) guidelines [20]. Since there is no correlation between *in vitro* susceptibility results for anti-tuberculosis agents such as rifampicin, ethambutol and rifabutin, susceptibility was evaluated on the basis of clarithromycin MIC only [2, 20]. All strains used in this study were found to be susceptible (MICs of ≤ 8 μg/mL).

All strains were initially cultured in 7H9 broth supplemented with 10% (vol/vol) OADC for 2 weeks at 37°C with shaking. The strains showed no apparent difference in growth and colony forming time on 7H10-OADC agar. Single-cell suspensions of cultured bacteria were prepared as previously described [21] and quantified by plating on 7H10-OADC agar. Briefly, mycobacterial cells grown in 7H9-OADC were harvested by centrifugation at 10,000 × *g* for 20 min, and then washed three times with 10 mM phosphate-buffered saline (PBS; pH 7.2). Mycobacterial cell pellets were homogenized using an overhead stirrer (Wheaton Instruments, Millville, NJ, USA) for 1 min on ice to minimize cell clumping. The homogenized mycobacterial cells were passed through an 8-μm pore size filter (Millipore Corp., Bedford, MA, USA). The presence of predominantly single cells in the final preparation was confirmed by acid-fast staining. Seed lots of each strain were stored in small aliquots at −80°C until use. Before conducting the infection study, ten-fold serial dilutions from seed lots were plated on 7H10 agar plates to quantify the number of organisms per milliliter.

### *In vivo* infection protocol

**Fig 1:**
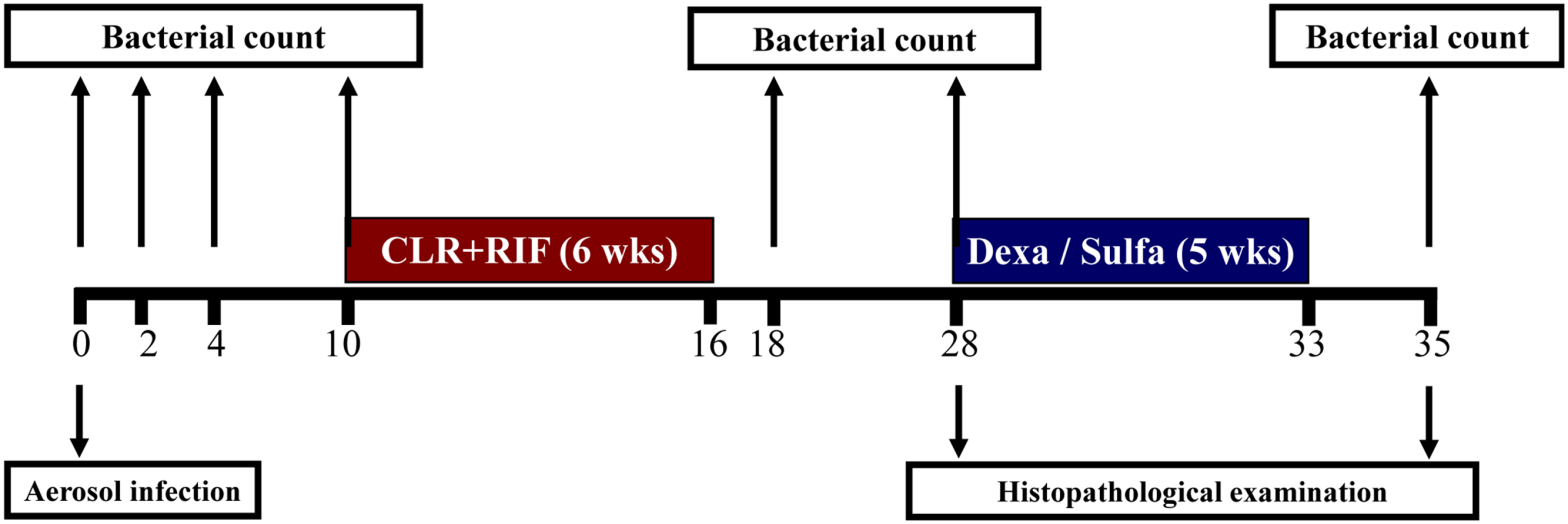
Schematic experimental procedure utilizing a modified Cornell-like mouse model. Mice were infected with each MAC strain by aerosol route. Ten weeks after infection, mice were treated with clarithromycin and rifampicin for six weeks. At 28 weeks post-infection, which was 12 weeks after completion of chemotherapy, mice received immunosuppressants for 5 weeks as described in Materials and Methods. Mice were euthanized and lungs were collected for bacteriological and histopathological examination at the time points depicted by arrows.

For the animal infection study, C57BL/6 mice were infected with each MAC strain via the Cornell model described by McCune *et al*. with minor modifications [22]. Briefly, each group of 6-week-old C57BL/6 mice was given an aerogenic infection with one of the MAC strain for 60 min in the inhalation chamber of an airborne infection apparatus (Glas-Col, Terre Haute, IN, USA) in a BL-3 biohazard animal room. Alternatively, control mice were inoculated with an equal volume of sterile PBS. For initial dose measurement, mice were killed one hour after infection and lungs were removed aseptically, homogenized, and plated. Five to six mice per group were euthanized at 2, 4, 10, 18, 28 and 35 weeks post-infection, and their lungs were collected for bacteriological and histopathological examinations (Fig 1).

### Treatment with antibiotics and immunosuppressants

A schematic of the experimental procedure used in this study is depicted in Fig 1. At 10 weeks post-infection, when stable infections of MAC were established in the lung, infected mice began to receive daily treatment with clarithromycin (200 mg/kg of body weight) and rifampicin (10 mg/kg of body weight) by oral gavage for a period of 6 weeks. Bacterial counts were taken from the lungs of mice at 2 weeks and again after completion of 6 weeks of chemotherapy to confirm clearance of cultivable organisms. At 28 weeks post-infection and, 12 weeks after the completion of chemotherapy, the treated mice received intraperitoneal injections of 200 μl of dexamethasone or sulfasalazine at 10 mg/kg of body weight every 2 days for 5 weeks (from 26 to 31 weeks post-infection). At 35 weeks post-infection and, 2 weeks after completion of immunosuppression therapy, the treated mice were euthanized and examined.

### Definition of “reactivation” and “regrowth”

The term “reactivation” refers to reactivation of latent TB. The definition of latent TB is evidence of infection such as a positive tuberculin skin test (TST) or IFN-γ release assay, without clinical symptoms or culturable bacilli in the sputum [23]. In this study, we used the term “reactivation” when the bacilli reappeared from a culture-negative state. Likewise, the term “regrowth” was used when there were countable bacilli after chemotherapy and relapse was observed after immune suppression.

### Bacterial counts

Five to six mice per group were euthanized at 2, 4, 10, 18, 28 and 35 weeks post-infection with CO_2_, and lungs were homogenized in a 0.04% Tween 80 saline solution using a tissue grinder (Wheaton, NJ, USA) with an automatic homogenizing stirrer (Daihan Scientific, Seoul, South Korea). The number of viable bacteria in the tissue suspension was determined by plating serial dilutions of the organ homogenates onto Middlebrook 7H11 agar supplemented with 10% OADC. Colonies were counted after incubation for 2 to 4 weeks at 37°C, and the limit of bacterial detection was determined as 25 CFU per whole lung after completion of antibiotic treatment (≥18 weeks).

### Histopathological analysis

For histopathological analysis, lung from the mice was excised and fixed for overnight in 10% normal buffered formalin. The tissues were trimmed into cassettes and embedded with paraffin. Each tissue blocks were cut into 4mm thick, and then mounted on microscopic slides. Sections were stained with hematoxylin and eosin (H&E) and examined for histopathological lesions. The level of inflammation in the lungs of the mice was evaluated using ImageJ software (National Institutes of Health, MD, USA), as previously described [24].

### Statistical analysis

The resultant values for bacterial counts were reported as the median ± interquartile range (IQR) and lung inflammation values were reported as the mean ± standard deviation (SD). The significance was determined by the Kruskal-Wallis test followed by Dunn’s test for bacterial count, and unpaired t-tests for inflammation values using statistical software (GraphPad Prism Software V5.0; GraphPad Software, San Diego, CA). Values of *P < 0.05, **P < 0.01 or ***P < 0.001 were considered to be statistically significant.

## Results

### Bacteriological evaluation of *Mycobacterium avium* complex reactivation

**Fig 2:**
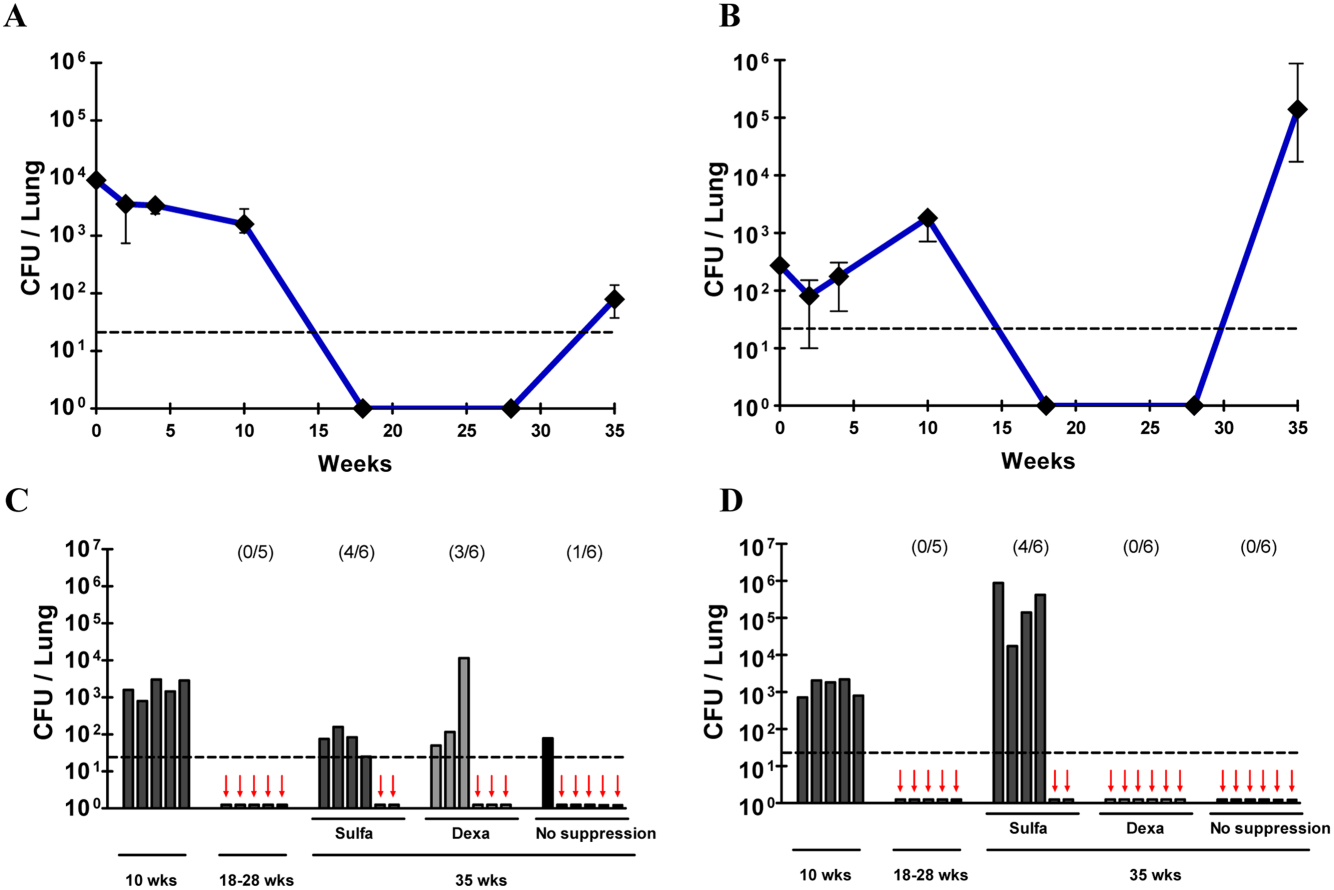
Reactivation of *Mycobacterium avium* complex in the lungs of mice following immunosuppression. Mice were infected with approximately 500–1,000 CFUs of each MAC strain for 10 weeks and treated for 6 weeks with clarithromycin and rifampicin. Following the antibiotic regimen, mice were treated with immunosuppressants. A: MAV SM#1 represents the reactivation model of *M*. *avium* strains. B: MI SM#42 represents the reactivation model of *M*. *intracellulare* strains. Both graphs depict a dramatic decrease in bacterial counts, which were undetectable after antibiotic treatment and a dramatic increase after sulfasalazine treatment. For A and B, CFUs at 35 weeks represent the sulfasalazine-treated group. C and D represent the bacterial counts of individual murine lungs infected with MAV SM#1 and MI SM#42, respectively. Each group exhibited different reactivation levels according to the immunosuppressant used. The dashed line represents the detection limit of bacterial counts. Red arrows represent undetectable bacilli in each mouse. For A and B, the data are the median ± interquartile range (IQR).

Bacterial counts of two clinical strains, MAV SM#1 and MI SM#42, decreased to an undetectable level after 18 weeks, which was after six weeks of antibiotics treatment (Fig 2A and 2B). Undetectable bacterial counts were maintained until the beginning of immunosuppressant treatment in all mice of these two groups. For the MAV SM#1 infected group, four out of six mice exhibited reactivation of bacteria after sulfasalazine treatment, and three out of six mice exhibited bacterial reappearance after dexamethasone treatment (Fig 2C). For the MI SM#42 infected group, four out of six mice had reappearance of bacterial counts after sulfasalazine treatment, and which were higher than that of the initial infection, whereas no mice had reappearance of bacteria after dexamethasone treatment (Fig 2D). One of the six mice infected with MAV SM#1 had bacterial reappearance without immunosuppression, suggesting the possibility of spontaneous reactivation. However, none of the MI SM#42 infected mice exhibited reactivation without immunosuppression. Interestingly, the reactivated bacterial count in MI SM#42 infected mice increased at a faster rate and was higher compared with the bacterial count before chemotherapy.

### Bacteriological evaluation of *Mycobacterium avium* complex regrowth

**Fig 3:**
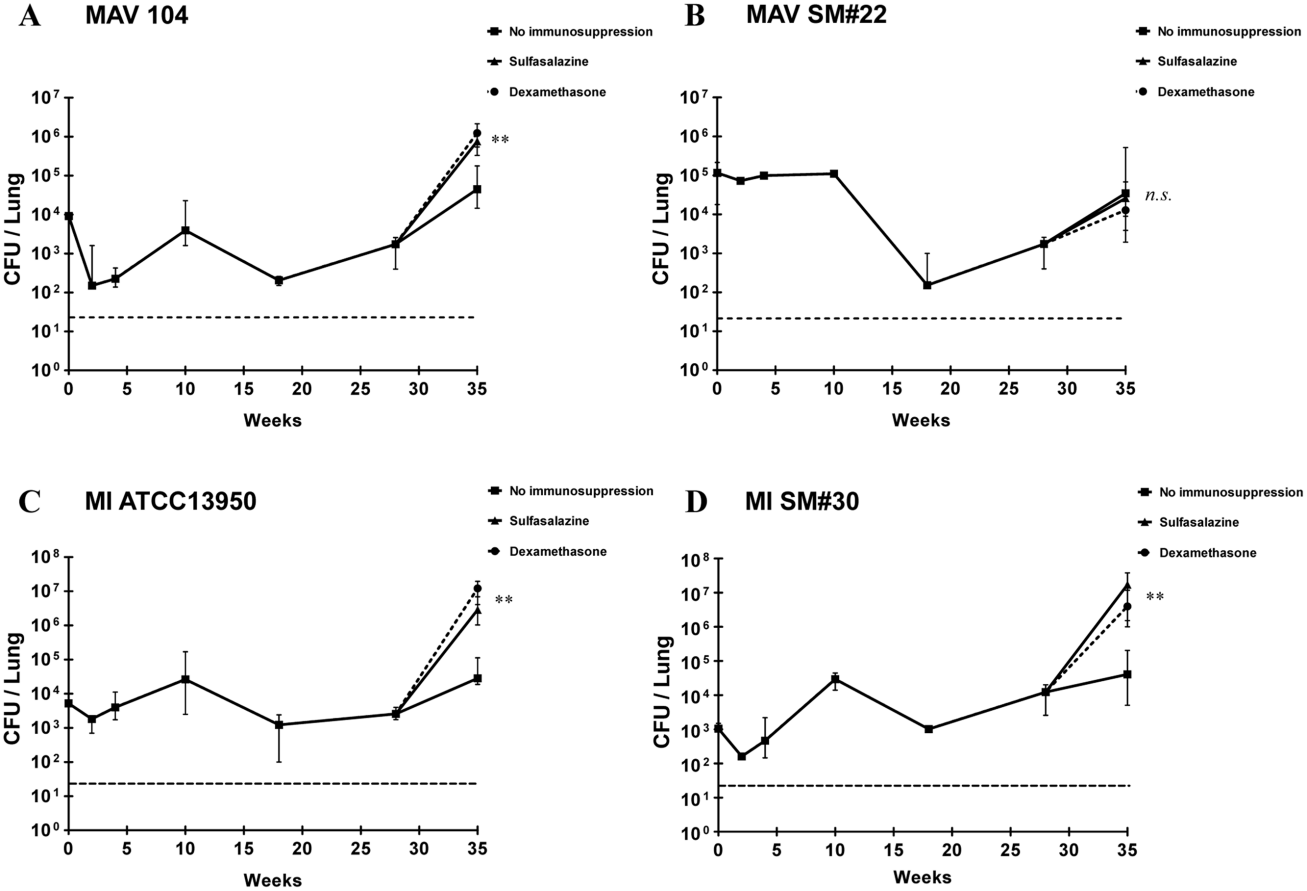
Regrowth of *Mycobacterium avium* complex in the lungs of mice following immunosuppression. A: MAV104 and B: MAVSM#22 are the regrowth models of *M*. *avium* strains. C: MI ATCC13950 and D: MI SM#30 are the regrowth models of *M*. *intracellulare* strains. All graphs show a dramatic decrease in bacterial counts, although they remained detectable after antibiotic treatment with clarithromycin and rifampicin. The number of bacterial counts showed dramatic increase after dexamethasone and sulfasalazine treatment. The dashed line represents the detection limit of bacterial counts. The data are the median ± interquartile range (IQR). **P < 0.01 compared to no immunosuppression group. *n*.*s*., not significant.

The bacterial counts of two *M*. *avium* strains, MAV 104 and MAV SM#22, were decreased at week 18 following 6 weeks of antibiotic treatment, but remained within detectable limits (Fig 3A and 3B). On the other hand, the bacterial counts at week 28 were slightly increased compared to the counts obtained prior to immunosuppressant treatment. The bacterial counts after immunosuppressant treatment exhibited a more rapid increase compare to MAV 104 infected mice that did not receive immunosuppression therapy (*P* < 0.01). Interestingly, there were no significant differences between the immunosuppressant-treated and untreated mice infected with MAV SM#22. The bacterial counts of the other two *M*. *intracellulare* strains, MI ATCC13950 and MI SM#30, showed similar patterns of regrowth as the *M*. *avium* regrowth model (Fig 3C and 3D). However, there was no significant difference in bacterial counts between the groups treated with sulfasalazine or dexamethasone.

### Histopathological evaluation of the *M*. *avium*-infected lungs

**Fig 4:**
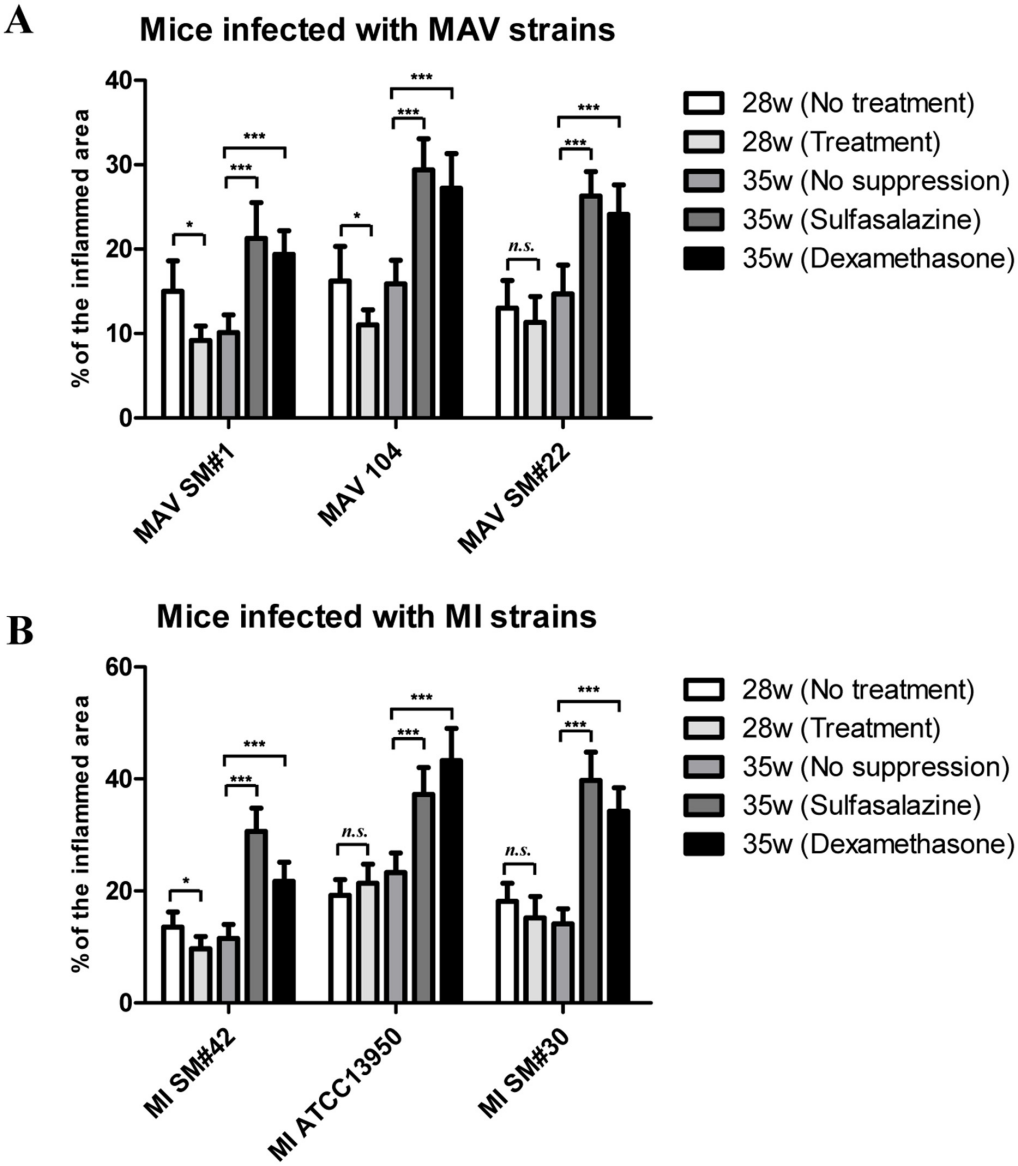
Lung inflammation values in mice. Lung inflammation values are presented as the mean percentage of the area of inflammation from lung infection of five to six mice per group (±standard deviation). At 28 weeks and 35 weeks post infection, lung samples from each mouse were analyzed as described in the Materials and Methods. The inflamed areas were significantly increased after immunosuppression. *P < 0.05, ***P < 0.001, *n*.*s*., not significant.

**Fig 5:**
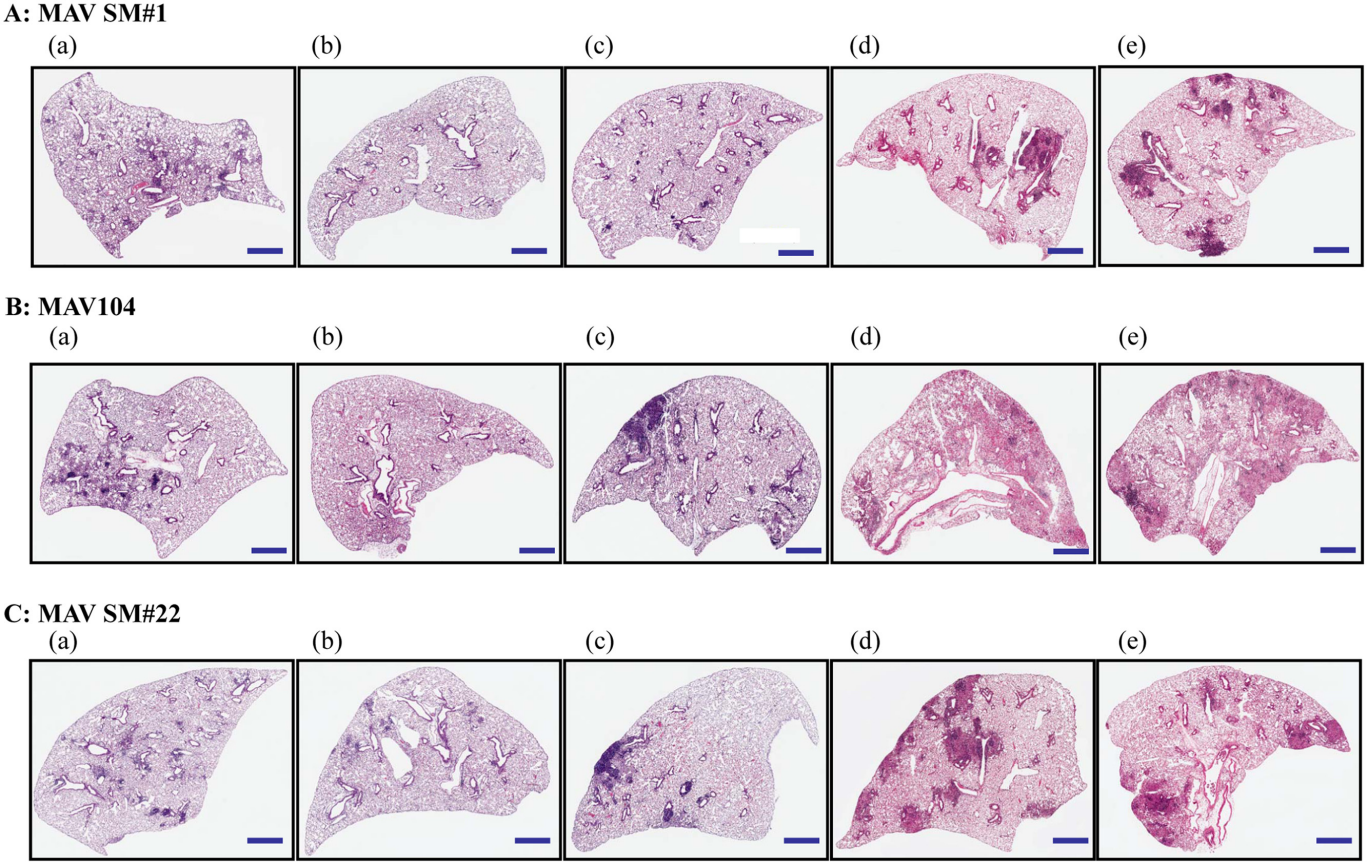
Histopathology of lungs in *M*. *avium*-infected mice. A: MAVSM#1 represents the reactivation model and B: MAV104 and C: MAVSM#22 are the regrowth models of *M*. *avium* strains. (a) Untreated controls and (b) antibiotic-treated samples at 28 weeks post infection. Histopathological changes of lungs with *M*. *avium* infection at 35 weeks post infection with no immunosuppression (c), sulfasalazine treatment (d), and dexamethasone treatment (e). The scale bar represents 1 mm.

Histopathological staining showed inflamed regions of *M*. *avium*-infected lungs. Specifically, H&E staining results showed an almost complete disappearance of inflammation was observed for the antibiotic treated lungs of MAV SM#1 infected mice at week 28 (Figs 4A and 5A), and a significant decrease (*P* < 0.05) inflammation with antibiotics-treated lungs compared to untreated lungs that were infected with MAV 104 (Figs 4A and 5B). The antibiotics-treated lungs that were infected with MAV 104 and MAV SM#22 showed reappearance of regions of inflammation without any further immunosuppressant treatment at week 35 and the magnitude of inflammation was significantly increased (*P* < 0.001) following treatment with either sulfasalazine or dexamethasone (Figs 4A, 5B and 5C). Interestingly, histopathology of MAV SM#22 infected lungs revealed significant inflammation after immune suppression, although the bacterial count in the lung remained the same. In the case of antibiotic treated lungs with previous MAV SM#1 infection, no significant occurrence of inflammation was detected without immunosuppressant treatments, while an obvious increase in inflammation was detected after immunosuppressant treatment (Figs 4A and 5A).

### Histopathological evaluation of the *M*. *intracellulare*-infected lungs

**Fig 6:**
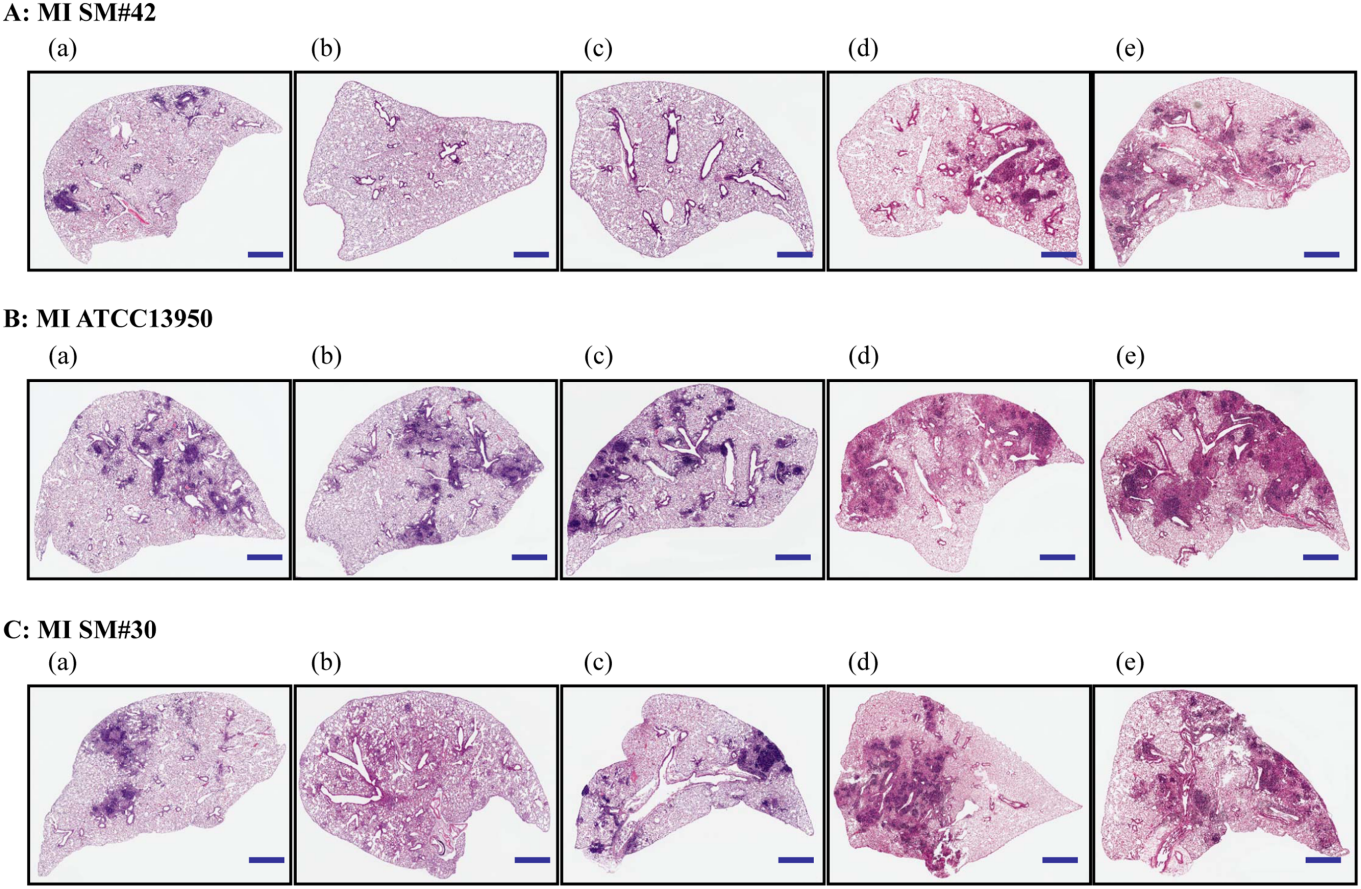
Lung Histopathology of *M*. *intracellulare* infected mice. A: MI SM#42 represents the reactivation model. B: MI ATCC13950 and C: MI SM#30 are the regrowth models of the indicated *M*. *intracellulare* strains. (a) Untreated controls and (b) antibiotic-treated samples at 28 weeks post infection. Histopathological changes of the lungs with *M*. *intracellulare* infection at 35 weeks post infection with no immunosuppression (c), sulfasalazine treatment (d), and dexamethasone treatment (e). The scale bar represents 1 mm at ×10 magnification.

Histopathological staining showed inflamed regions of *M*. *intracellulare*-infected lungs (. The antibiotics-treated lung with previous MI SM#42 infection did not exhibit reoccurrence of inflammation without immunosuppressant treatment; however, significantly increased (*P* < 0.001) inflammation was detected after immunosuppressant treatment (Figs 4B and 6A). H&E staining results revealed no significant decreases of inflammation regions in the antibiotics-treated lungs compared to untreated lungs infected with MI ATCC13950 and MI SM#30. On the other hand, inflammation in the antibiotic treated lungs infected with MI SM#42 was almost completely absent at week 28 (Figs 4B and 6A). The infection of MI ATCC13950 and MI SM#30 lungs treated with antibiotics showed continuous regions of inflammation without further treatment at week 35, and the magnitude of inflammation was significantly increased (*P* < 0.001) compared to the initial infections after treatment with sulfasalazine or dexamethasone (Figs 4B, 6B and 6C).

## Discussion

LTBI, a primary concern in the treatment of TB, has been investigated by numerous researchers using various animal models. However, there have been few studies on latent MAC infections, even though the symptoms of pulmonary MAC infection are quite analogous to those of TB. Evidence for the existence of MAC recurrence is relatively recent, and its pathogenesis has not been studied as well as that of TB. Recently, there have been several case reports suggesting the possibility of MAC reactivation based on the genomic identity between initial isolates and recurrent isolates after chemotherapy [25, 26]. In addition, a population-based national survey in the United States revealed that positive result rates for MAC skin tests peak in young adults and decrease in older populations [7]. Nevertheless, MAC-associated lung disease is generally considered to be most common in the elderly [1, 2]. Researchers have speculated that these findings may be related to the existence of a latency as well as emaciating of the immune system with age, which could explain both the lower positive rate of MAC skin test and the higher rate of NTM-related lung disease [7]. Likewise, the development of NTM-related lung disease shortly after TNF antagonist treatment has provided further evidence for the possibility of latent MAC infection [9–11]. However, it has not yet been confirmed experimentally whether or not MAC infection can enter a latent state. In light of this current state, we hypothesized that MAC can cause latent infection, and confirmed this hypothesis by observing reactivation of MAC infections in the Cornell-like murine model [14, 15].

In the present study, bacterial count results and histopathological analysis of lungs suggested that the reactivation of MAC can occur under a compromised immune system (systemic immunosuppression). MAV SM#1- and MI SM#42-infected mice appeared to be successfully treated with antibiotics since the bacteria counts were decreased to an undetectable level. However, most of the mice, that were thought to have undergone bacterial clearance presented with a dramatically increased number of bacterial counts after immunosuppressant treatment. Although there were differences in the rate of reactivation depending on bacterial strain and type of immunosuppressant, it is quite reasonable to assume that the bacteria had gone into a latent phase and were reactivated following compromise of the immune system, since there were no other sources of mycobacterial infection in these mice. In addition, our results suggest a model of regrowth for some MAC strains that do not respond well to antibiotics typically used for MAC-associated disease. Indeed, the remaining bacteria in some animals exhibited significant regrowth after antibiotic treatment following immunosuppressant treatment, exhibiting a faster rate of regrowth compared to immunocompetent animals (Fig 3).

There are no reported standardized protocols for establishing latency of TB within the Cornell model, and outcomes can vary depending on experimental parameters such as the mycobacterial strain used, inoculating dose, route of infection, infection period, concentration of antibiotics, length of treatment and the post-antibiotic rest periods [27]. Thus, we employed several clinical isolates of MAV and MI in this study. As expected, the different strains exhibited different *in vivo* growth kinetics although they showed similar growth kinetics *in vitro*. This difference in *in vivo* growth kinetics, which might reflect differences in host-pathogen interactions, may have affected responses to the same antibiotic treatment. As a result, we were able to define two groups of reactivation and regrowth in terms of presence of cultivable bacilli after treatment with antibiotics. Macrolides are primarily recommended as part of a therapeutic regimen for MAC lung disease [2]. Although rifampicin is known to affect the metabolism of clarithromycin resulting in decreased serum concentrations of the latter [28], we selected clarithromycin and rifampicin according to the standardized combination antibiotic therapy used in human MAC disease [2]. However, in this study we failed to induce a culture-negative state for two strains of MAC. We were unable to clarify the concentration of antibiotics and the length of treatment for the sterilized MAC due to a lack of reference studies using this combination of antibiotics in a mouse model followed by aerosol infection. Thus, it is possible that these factors may have contributed to different rates of bacterial clearance after antibiotic treatment.

With respect to the route of infection, we chose an aerosol infection route, which has been shown to facilitate bacterial colonization in the lung and subsequent proliferate within the tissue [29]. While infection through the intravenous route elicits a systemic immune response in the host and is less pathogenic in a murine TB model [30], pathologic lung lesions do not appear in murine *Mycobacterium abscessus* models [31]. As we aimed to induce lung pathologic lesions corresponding to bacterial load at different time points in this study, we chose an aerosol infection route. We found that most of the mice infected by aerosol route with MAC did not exhibit bacterial dissemination to spleen by 10 weeks post-infection (prior to antibiotics treatment), although similar patterns of MAC regrowth in the spleen and lungs were observed after immunosuppression (S1 and S2 Figs). Thus, we focused on bacterial burden and histopathology of the lungs which is the organ most remarkably affected by MAC aerosol infection.

In this study, we used dexamethasone and sulfasalazine for immune suppression. Dexamethasone is a glucocorticoid immunosuppressant that is commonly used to induce reactivation of latent TB in Cornell-like animal models. However, to the best of our knowledge, this study is the first use of sulfasalazine as an immunosuppressant for mycobacterial reactivation. Interestingly, sulfasalazine-treated mice showed a higher reactivation rate compared to dexamethasone-treated mice with 66.7% and 50.0% observed for MAV SM#1 infection, and 66.7% and 0% for MI SM#42 infection, respectively. As these two immunosuppressants have different mechanisms of action, further investigation of the differences in immunological events after treatment with these agents may provide clues for detailed immunological mechanisms of reactivation for the different MAC strains.

In spite of the variations observed for the Cornell-like murine model, we successfully removed the bacteria in at least one strain of each MAC species by antibiotic treatment and induced reactivation after immune suppression. Since MAC are ubiquitously distributed in the environment and are frequently encountered by individuals who receive skin tests or receive TNF antagonist therapy, the skin test results or TNF antagonist treatment cannot be considered direct evidence of latent MAC infection. In addition, a previous study demonstrated latent MAC infection in simian immunodeficiency virus-infected rhesus macaques [32]. However, the findings of that study were limited by the fact that the monkeys were housed outdoors and the source of infection was not identified [32]. To avoid this limitation, we fully controlled all sources of MAC infection during our study except for the first intentional aerosol infection, and achieved the reactivation of MAC after immune suppression followed by chemotherapy. Therefore, reactivation from a latent state of MAC infection is possible explanation for our observation. Moreover, our data showed that the rapid regrowth of MAC in immunocompromised mice was consistent with the general concept that the host immune systems are important in controlling MAC infection.

The Cornell murine model was originally adopted to understand some aspects of latency and reactivation of TB after these processes were firstly and clinically proven in humans. Here we employed this Cornell-like murine model to investigate the reactivation of MAC, since whether or not MAC bacilli exist in a truly latent form in humans remains an open question. One recent clinical study reported that reinfection with new MAC genotypes is a common cause of recurrence of MAC infection [33] and that a quarter of recurrences were true relapses after completion of therapy. Although it is extremely difficult to distinguish whether cases of true relapse resulted from reinfection with the same genotype or from reactivation of latent infection, our study experimentally presented the possibility of endogenous reactivation of MAC from a latent state due to fundamental blockade of other sources of MAC infection. Nevertheless, as the latency and reactivation of MAC has not yet been verified in a natural host, our study has several limitations. Specifically, lack of additional evidence of a latent state such as changes in gene expression in MAC and changing patterns of cytokine expression in the host should be investigated in future studies. Moreover, detection of latent state MAC by the plating assay may not have been ideal for cultivating antibiotic-injured bacteria or viable but non-culturable bacteria. To differentiate between undetectable bacilli and non-culturable states it might be useful to verify the presence of bacterial RNA in apparently sterilized tissues after completion of antibiotic treatment. Nevertheless, TST is still used as a standard method for diagnosing latent TB as nucleic acid amplification tests or acid-fast staining of bacilli are not recommended for diagnosis of latent TB because of their low sensitivity [34, 35]. In the case of TB, the latency was first clinically verified and has been studied widely in experimental settings such as the Cornell model. Although MAC latency and reactivation is not clinically established, the potential for MAC reactivation could not be excluded based on our experimental results.

In summary, our study highlights the possibility for relapse of MAC pulmonary infection resulting from reactivation of a latent infection. In addition, our results demonstrate that an immune-compromised status can serve as a key trigger to induce reactivation of latent MAC infection in an experimental mouse model.

## Supporting Information

S1 Fig Reactivation of *Mycobacterium avium* complex in the spleens of mice following immunosuppression. Mice were infected with approximately 500–1,000 CFUs of each MAC strain for 10 weeks and treated for 6 weeks with clarithromycin and rifampicin. Following the antibiotic regimen, mice were treated with immunosuppressants. A and B represent the bacterial counts of individual murine spleens infected with MAV SM#1 and MI SM#42, respectively. Dashed line represents the limit of detection. Red arrows represent undetectable bacilli in each mouse. For A and B, the data are the median ± interquartile range (IQR).
(TIF)

S2 Fig Regrowth of *Mycobacterium avium* complex in the spleens of mice following immunosuppression. A: MAV104 and B: MAVSM#22 are the regrowth models of *M*. *avium* strains. C: MI ATCC13950 and D: MI SM#30 are the regrowth models of *M*. *intracellulare* strains. Dashed line represents the limit of detection. The data are the median ± interquartile range (IQR). **P < 0.01 compared to no immunosuppression group. *n*.*s*., not significant.
(TIF)

S1 File NC3Rs ARRIVE Guidelines Checklist. (PDF)
