# Supplementary figures and images for: Experimental Reactivation of Pulmonary Mycobacterium avium Complex Infection in a Modified Cornell-Like Murine Model
Source: PLoS One. 2015 Sep 25;10(9):e0139251. doi: 10.1371/journal.pone.0139251 (PMC4583228; doi:10.1371/journal.pone.0139251)

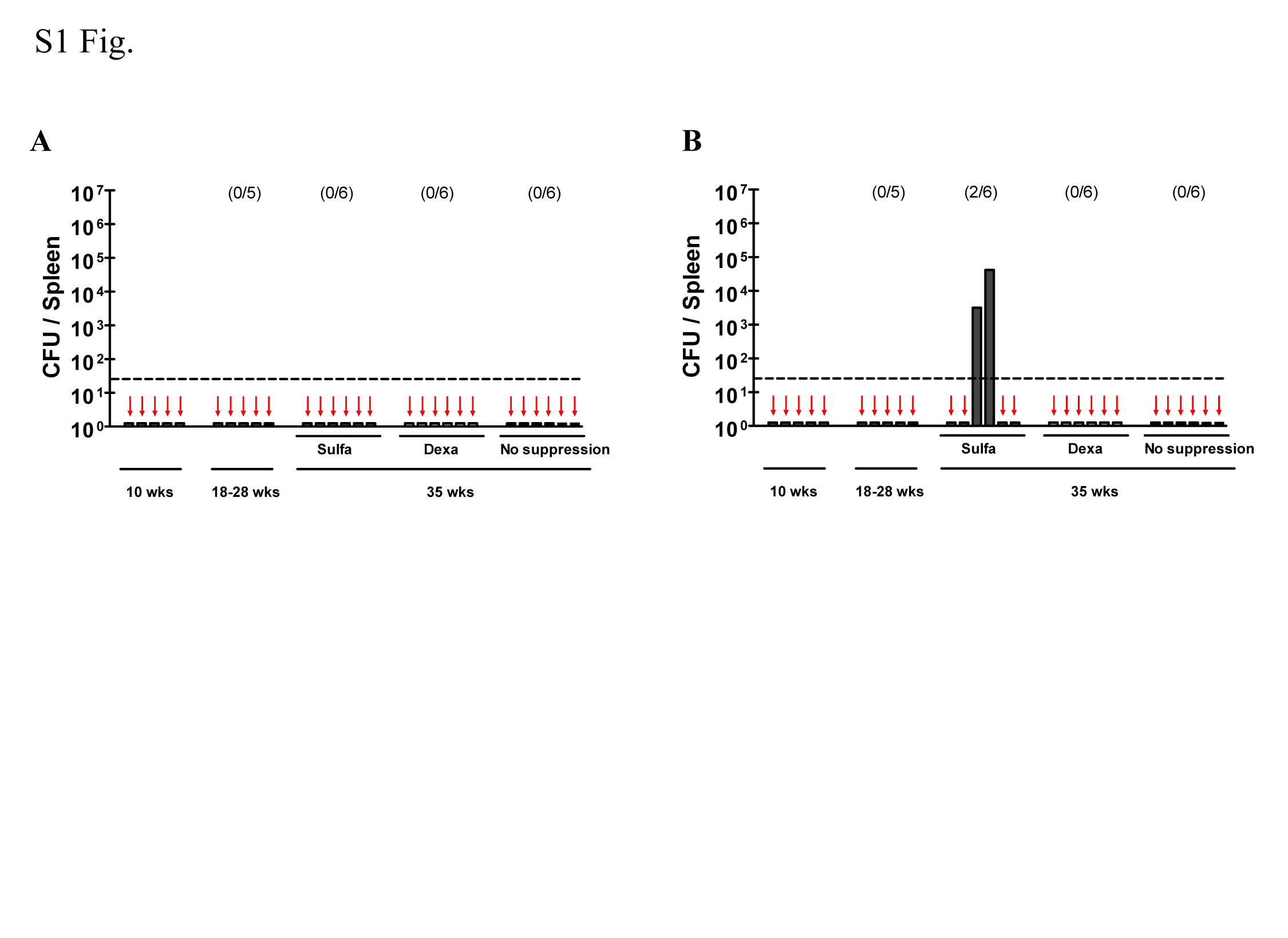

Supplement: S1 Fig — Mice were infected with approximately 500–1,000 CFUs of each MAC strain for 10 weeks and treated for 6 weeks with clarithromycin and rifampicin. Following the antibiotic regimen, mice were treated with immunosuppressants. A and B represent the bacterial counts of individual murine spleens infected with MAV SM#1 and MI SM#42, respectively. Dashed line represents the limit of detection. Red arrows represent undetectable bacilli in each mouse. For A and B, the data are the median ± interquartile range (IQR). (TIF) [file pone.0139251.s001.tif]

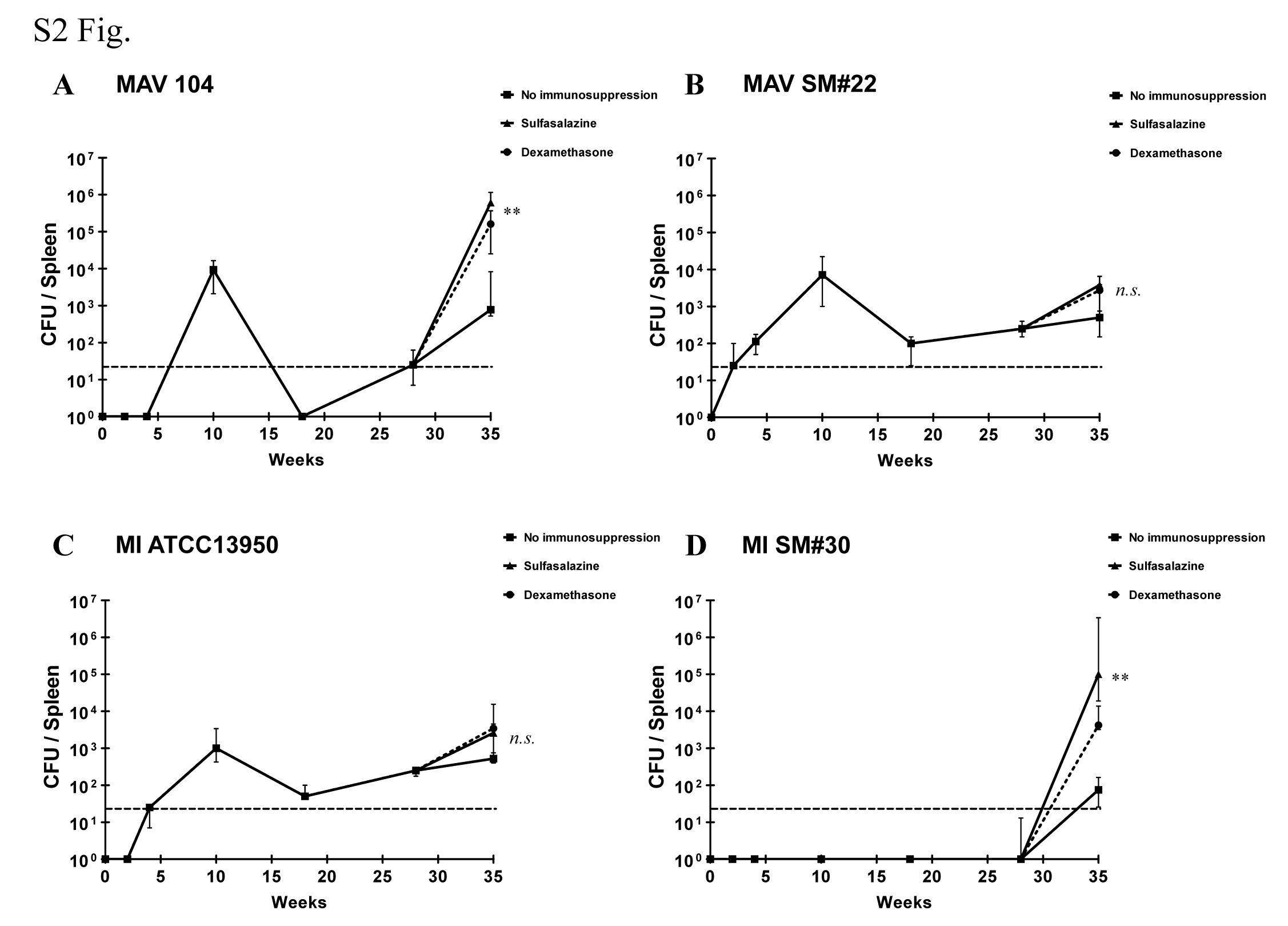

Supplement: S2 Fig — A: MAV104 and B: MAVSM#22 are the regrowth models of M. avium strains. C: MI ATCC13950 and D: MI SM#30 are the regrowth models of M. intracellulare strains. Dashed line represents the limit of detection. The data are the median ± interquartile range (IQR). **P < 0.01 compared to no immunosuppression group. n.s., not significant. (TIF) [file pone.0139251.s002.tif]
